# Comparison of regional and global cardiac MRI diastolic strain rates with echo grading of diastolic dysfunction

**DOI:** 10.1186/1532-429X-18-S1-P193

**Published:** 2016-01-27

**Authors:** Tarun Pandey, Mohan Mallikarjuna Rao Edupuganti, Alapati Sindhura, Shelly Lensing

**Affiliations:** 1 Radiology, University of Arkansas for Medical Sciences, Little Rock, AR USA; 2 Cardiology, University of Arkansas for Medical Sciences, Little Rock, AR USA; 3 Biostatistics, University of Arkansas for Medical Sciences, Little Rock, AR USA

## Aim

To study use of feature tracking technique in evaluation of regional and global diastolic strain rate and to define the best predictor of diastolic dysfunction by studying its correlation with echo grading of diastolic dysfunction.

## Background

Echocardiographic examination is currently the gold standard for the non invasive assessment of diastolic dysfunction. Although strain is not load independent, studies have shown that systolic strain rates can assess myocardial contractility and are relatively load independent. It remains to be established if diastolic strain parameters correlate with the degree of diastolic dysfunction and if so which parameter correlates best with echo derived grade of diastology.

## Methods

We performed a retrospective, IRB approved, review of 46 patients who underwent cardiac MRI at our facility for evaluation of cardiac amyloidosis. All patients had an echocardiographic assessment of diastolic function (grades 0, 1, 2 and 3). Conventional short axis, vertical long axis and 4-chamber cine SSFP (Single shot Free Precession) images from the cardiac MRI scans were used to generate 2D (radial, circumferential) and 3D (longitudinal) diastolic strain rate maps using myocardial feature tracking software. The diastolic strain rates were compared with echocardiographic grades of diastology.

## Results

Patients had an average age of 61.5 years; males 61%, 17 patients had grade 0, 16 had grade 1 and 13 had grade 2/3 diastolic dysfunction. Base level parameters had the strongest correlations with diastolic dysfunction. The strongest correlations were found for base radial 2D (Spearman's rank correlation, 0.46, p = 0.002) and longitudinal 3D diastolic (-0.46, p = 0.001) diastolic strain rates. Across all levels on average, radial 2D diastolic strain rate had the strongest correlation with diastolic dysfunction (0.46) followed by circumferential 3D (-0.36) and longitudinal 3D (-0.36) diastolic strain rates.

Also, the base diastolic parameters separated best according to echo grade of diastolic dysfunction: The base longitudinal 3D differed the most between grade 0 vs. all grades of diastolic dysfunction (median 122.74, 93.12 and 73.03 respectively for grades 0, 1, 2/3; Respective p values were p = 0.001, 0.003 and 0.008). The radial 2D diastolic strain rate differed the most between grades 1 vs. Grades 2/3 (median 216.21 vs. -136.14, p = 0.036. Mid ventricular radial 2D strain rate had the most significant difference between grade 1 vs. grade 2/3 diastolic dysfunction (Median, -156.73 vs. -126.93, p = 0.019).

## Conclusions

MRI feature tracking is a useful tool for detection of diastolic dysfunction. Our study demonstrated that global radial 2D strain rates and global longitudinal and circumferential 3D diastolic strain rates correlate best with the degree of diastolic dysfunction. For segmental analysis, measuring basal 2D and longitudinal 3D diastolic strain rates will provide the best measure of diastology.

